# Visual inputs and postural manipulations affect the location of somatosensory percepts elicited by electrical stimulation

**DOI:** 10.1038/s41598-019-47867-1

**Published:** 2019-08-12

**Authors:** Breanne P. Christie, Hamid Charkhkar, Courtney E. Shell, Paul D. Marasco, Dustin J. Tyler, Ronald J. Triolo

**Affiliations:** 1 Department of Biomedical Engineering, Case Western Reserve University, Cleveland, OH USA; 2 Louis Stokes Cleveland Department of Veterans Affairs Medical Center, Cleveland, OH USA; 3 Department of Biomedical Engineering, Lerner Research Institute, Cleveland Clinic, Cleveland, OH USA

**Keywords:** Biomedical engineering, Somatosensory system, Somatic system

## Abstract

The perception of somatosensation requires the integration of multimodal information, yet the effects of vision and posture on somatosensory percepts elicited by neural stimulation are not well established. In this study, we applied electrical stimulation directly to the residual nerves of trans-tibial amputees to elicit sensations referred to their missing feet. We evaluated the influence of congruent and incongruent visual inputs and postural manipulations on the perceived size and location of stimulation-evoked somatosensory percepts. We found that although standing upright may cause percept size to change, congruent visual inputs and/or body posture resulted in better localization. We also observed visual capture: the location of a somatosensory percept shifted toward a visual input when vision was incongruent with stimulation-induced sensation. Visual capture did not occur when an adopted posture was incongruent with somatosensation. Our results suggest that internal model predictions based on postural manipulations reinforce perceived sensations, but do not alter them. These characterizations of multisensory integration are important for the development of somatosensory-enabled prostheses because current neural stimulation paradigms cannot replicate the afferent signals of natural tactile stimuli. Nevertheless, multisensory inputs can improve perceptual precision and highlight regions of the foot important for balance and locomotion.

## Introduction

Over two million people are living with limb loss in the United States^1^. Commercially available prostheses offer various control mechanisms (manual, body-powered, myoelectric) and multiple degrees of freedom, however none currently provide their users with somatosensory feedback. Previous studies have added sensory feedback via transcutaneous electrical stimulation^2–7^, vibration^6,8–11^, and by directly interfacing with the nerve^12–16^. Adding this feedback can improve functional ability^10,14,15,17–22^, reduce phantom pain^2,14^, and enhance prosthesis embodiment (i.e., incorporation of a prosthesis into one’s body schema)^18,23–26^. It is less clear how such somatosensory feedback integrates with other inputs, such as visual information and body posture, to shape one’s perception of the environment. In order to develop maximally beneficial somatosensory neuroprostheses, the impact of these inputs on elicited somatosensory feedback needs to be better understood.

The connection between natural tactile somatosensation and vision is strongly demonstrated in psychophysical and biological studies. Previous psychophysical studies have shown that tactile spatial resolution improves by adding vision^27–29^. Electrophysiology experiments have supported these claims: viewing the body modulates human primary somatosensory cortex activity^30,31^, and tactile stimulation enhances activity in the visual cortex^32^. Improvements in tactile acuity occur even when vision of the tactile stimulus is non-informative, i.e., an individual views the body part that is touched but does not see the tactile stimulus itself^28,29,33–36^. Tactile and visual feedback are thought to be integrated and inversely weighted by the uncertainty associated with each feedback modality; that is, the modality with greater uncertainty is weighted less^27^. As long as conditions for visual inputs are favorable, such as when there is sufficient lighting and contrast, uncertainty is typically lower for visual feedback compared to touch, leading to greater trust in visual feedback^27^. Subsequently, estimates of the environment are more accurate with feedback from both touch and vision than estimates from either modality alone^27^.

When somatosensory and visual information are spatially incongruent, the parietal cortex is assumed to attempt to reestablish congruency by modulating the “gain” of sensory systems^37^. When using mirrors to introduce conflict between the vision of touch and the feeling of touch, tactile sensitivity increases. If transcranial magnetic stimulation is applied to temporarily disengage the posterior parietal cortex, the gaining mechanism is temporarily eliminated. A visible consequence of this corrective gain is the phenomenon of visual capture: when visual and tactile inputs do not occur in the same location, somatosensory percepts can shift towards the location of visual inputs^38^. Once the multimodal mismatch is too large, however, the gaining mechanism is not sufficient and two inputs are no longer perceived as spatially congruent. Despite the well-studied relationship between somatosensation and vision, it is not clear whether the same connection still holds for prostheses with added sensory feedback.

The nervous system also utilizes postural information to determine the location of touch^39–42^. Information about the location of a tactile stimulus on the surface of the skin is combined with proprioceptive information about the location of each part of the body^43^. Most prior studies examined the relationship between posture and tactile localization by asking participants to cross their arms^42^ or turn their heads^44^. Posture manipulations that occur during locomotion, such as when the heel strikes the ground, have not been explored with respect to tactile localization.

Additionally, cognitive expectations arise from our internal knowledge of body posture. Prior experiences tell us that while standing upright, we expect to feel our feet touching the ground. These expectations of what we *should* feel influence what we *do* feel^45^. This is illustrated by a previous study that measured event-related potentials (ERPs) using electroencephalography (EEG) during a self-generated movement task with human participants. Participants were instructed to move their hand to touch their chest. Before initiating the movement, a task-irrelevant tactile probe was applied to their chest. They found that action preparation modulated tactile probe-evoked somatosensory ERPs^46^. Our cognitive expectations largely influence how we perceive reality, which is also demonstrated by illusions resulting from differences between our perception and physical reality. Illusions can achieve a desired perceptual effect by compensating for missing information with the remaining senses^47^. For example, in one prior study, providing visual information about the stiffness of a virtual spring resulted in reports of haptically feeling physical resistance^48^.

To further explore the roles of visual information and posture in tactile localization, we utilized peripheral nerve stimulation (PNS) via implanted nerve cuff electrodes to disassociate multisensory stimuli. We have previously demonstrated that the electrical activation of residual peripheral nerves of trans-tibial amputees can generate somatosensory percepts projected to the missing feet^13^. In this study, we tested scenarios of congruent and incongruent visual inputs and postural manipulations to determine how multisensory integration affects stimulation-induced somatosensory perception. We hypothesized that changing body position from seated to standing would not impact percept size and location, that congruent information would confine percepts, and that incongruent information would cause percepts to spread. We anticipated that stimulation-induced tactile percepts with locations irrelevant to locomotion (such as tactile sensation on the side of the ankle) could become more functionally relevant as a result of visual inputs and postural manipulations.

## Materials and Methods

### Research participants

Two volunteers with unilateral trans-tibial amputations (LL01 & LL02) due to trauma were enrolled in this study. At the time of device implantation, LL01 was 67 years old and had lost his limb 47 years prior. LL02 was 54 years old and lost his limb nine years beforehand. Both participants were male, regular prosthesis users, and did not have peripheral neuropathy or uncontrolled diabetes. The Louis Stokes Cleveland Veterans Affairs Medical Center Institutional Review Board and Department of the Navy Human Research Protection Program approved all procedures. This study was conducted under an Investigational Device Exemption obtained from the United States Food and Drug Administration. Both participants gave their written informed consent to participate in this study, which was designed in accordance with relevant guidelines and regulations.

### Implanted technology and delivery of electrical stimulation

**Figure 1:**
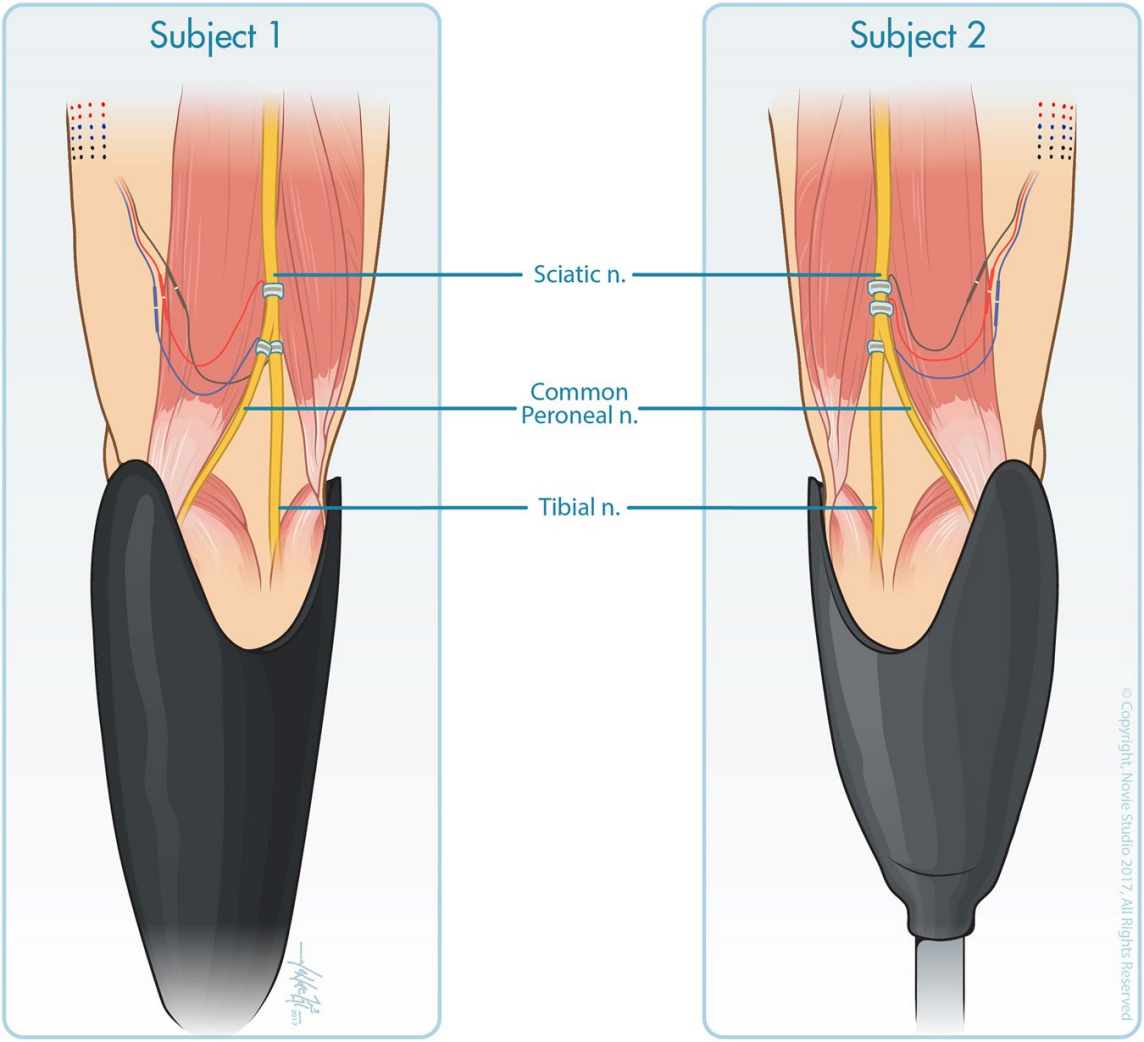
Three 16-contact C-FINEs were implanted around the sciatic, tibial, and common peroneal nerves of subject LL01 (left) and around the proximal sciatic, distal sciatic, and tibial nerves of subject LL02 (right). Reproduced from Charkhkar *et al*.^13^, (10.1088/1741-2552/aac964). © IOP Publishing Ltd. CC BY 3.0. Also printed with permission from © *Novie Studio*.

Both participants had 16-contact Composite Flat Interface Nerve Electrodes (C-FINEs)^49^ installed around their sciatic, tibial and/or common peroneal nerves (Fig. 1)^13^. The details of implant procedure and post-operative care are described in our prior work^13^. Both participants received implants in 2016. The described experiments in this study were performed at least one year post-implantation, and participants received electrical stimulation near weekly during other experiments prior to this study. All C-FINE contacts were connected to percutaneous leads that exited the skin on the upper anterior thigh. These percutaneous leads were connected to a custom-designed external stimulator that had a maximum stimulation amplitude of 5.6 mA and a maximum pulse width of 255 μs^50,51^. Stimulation waveforms were monopolar, asymmetric biphasic, charge-balanced, cathodic-first pulses with return to a common anode placed on the skin above the ipsilateral iliac crest. Stimulation parameters were set in MATLAB (MathWorks, Inc.; Natick, MA, USA) and then sent to a single board computer running xPC Target (MathWorks, Inc.; Natick, MA, USA), which controlled the external stimulator in real time. Stimulation was limited to a charge density of 0.5 μC/mm^2^ in order to minimize the risk of tissue and/or electrode damage^52^.

### Experimental design

Participants were instructed to adopt a specific posture, as described below, while electrical stimulation was delivered for five consecutive seconds to selected electrode contacts. When stimulation ended, participants drew the location of the elicited percept on a blank diagram of a generic healthy foot. The drawing was captured electronically using a touchscreen display (Cintiq 27QHD Touch; Wacom Co., Kazo, Saitama, Japan).

**Figure 2:**
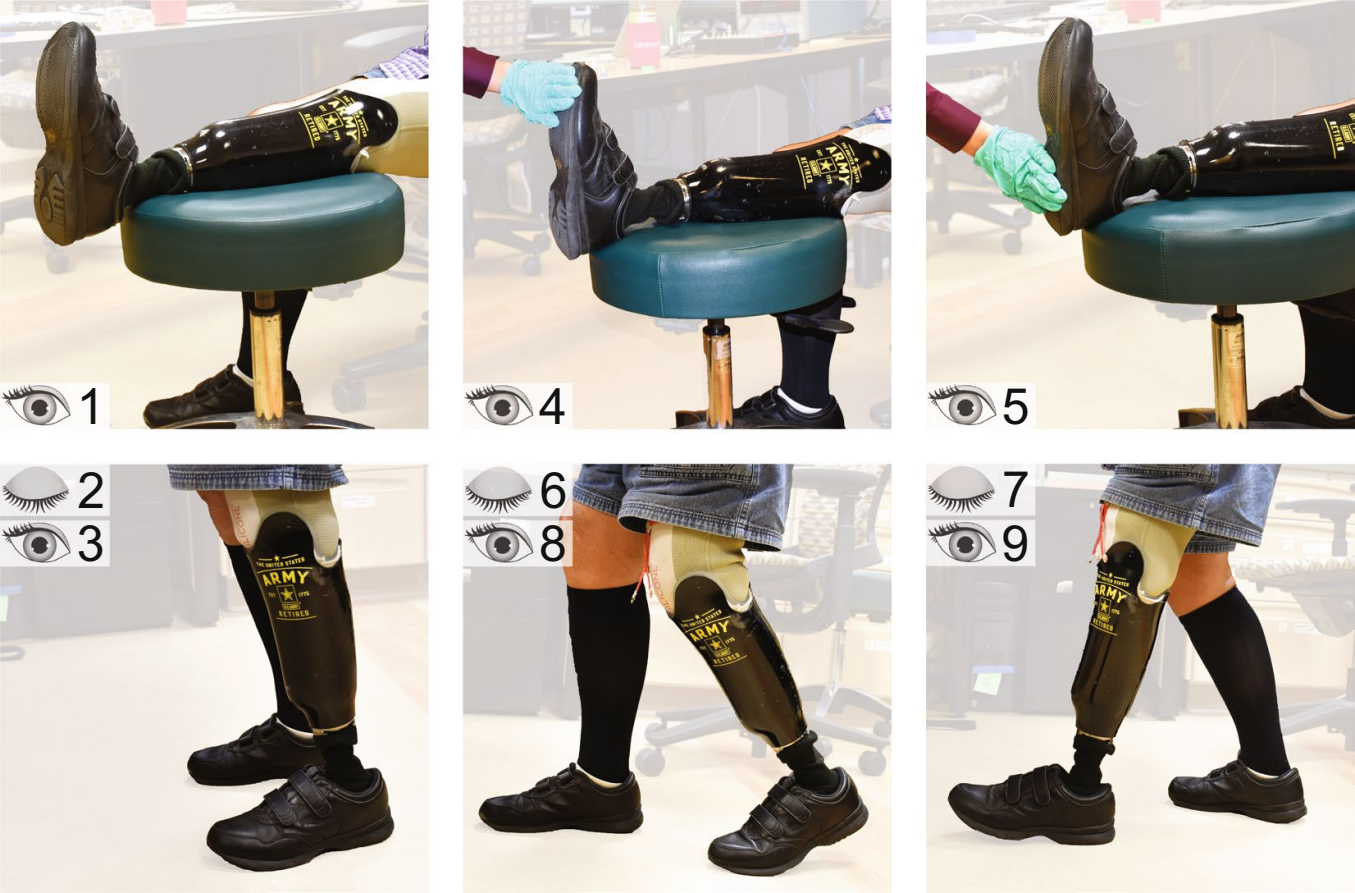
Experimental conditions are demonstrated by participant LL01. The number(s) in the left corner of each photo represent the condition number described in Table 1. The three photos in the bottom row have two numbers because each posture was repeated with the eyes open and closed.

In the baseline condition, participants sat down and placed the prosthetic foot on a stool such that the knee was fully extended (#1 in Table 1 and Fig. 2). The dorsal surface of the prosthetic foot was in clear view and the participants were instructed to look at it. In static standing conditions, the participants stood upright with their eyes closed (#2) or with their eyes open and looking down at the prosthesis (#3). When standing upright, there was foot-floor interaction due to weight bearing. While standing upright with the eyes open, there was also visual confirmation of this prosthetic foot-floor contact.

**Table 1:** Summary of the body position, visual inputs, and postural manipulations involved in each experimental condition. Electrical stimulation was combined with each condition.

Condition		Body Position	Visual Inputs	Postural Manipulations
#1	Baseline	Seated	None	None
#2	Static standing	Standing	None	None
#3			Observation of prosthetic foot-floor contact	None
#4	Visual input	Seated	Observation of experimenter lightly touching the plantar surface of the prosthetic **forefoot**	None
#5			Observation of experimenter lightly touching the plantar surface of the prosthetic **rearfoot**	None
#6	Posture manipulation	Standing	None	Adopting a posture that applies a load on the plantar surface of the prosthetic **forefoot**
#7			None	Adopting a posture that applies a load on the plantar surface of the prosthetic **rearfoot**
#8	Visual input + posture manipulation		Observation of prosthetic foot-floor contact	Adopting a posture that applies a load on the plantar surface of the prosthetic **forefoot**
#9			Observation of prosthetic foot-floor contact	Adopting a posture that applies a load on the plantar surface of the prosthetic **rearfoot**
#S1	Static standing, prosthesis off	Standing with no prosthesis	None (contacts R2 and R3)	None
			Eyes open, looking ahead (contacts F1 and F2)	
#S2	Static standing, prosthesis on but unloaded	Standing with the intact leg on a wooden box and the prosthetic leg dangling in the air (“unloaded”)	None (contacts R2 and R3)	None
			Eyes open, looking ahead (contacts F1 and F2)

During conditions with added visual inputs, the participants remained seated while watching an experimenter lightly touch the prosthetic plantar forefoot (#4) or rearfoot (#5). The forefoot encompassed the toes and the metatarsals, and the rearfoot encompassed the heel. The experimenter followed real-time visual cues on a computer screen (visible only to the experimenter and not to the participant) to determine the onset and offset for applying touch. The visual cues on the screen were synchronized with the timing of electrical stimulation delivery. The manner of applied physical touch was a mild constant pressure to the bottom of the shoe on the prosthetic foot. The touch was applied such that participants did not feel any added pressure through their socket and could therefore not detect the touch if their eyes were closed. The plantar surface of the foot, where the physical touch was applied, was not visible. This case of non-informative vision was intentional, given that the plantar surface of the foot is typically not visible during locomotion.

During conditions with postural manipulations, the participants stood upright with their eyes closed and adopted a posture that applied a load on either the plantar surface of the prosthetic forefoot (#6) or rearfoot (#7). During conditions with postural manipulations and visual inputs, participants repeated the same postures while looking down at the prosthesis with their eyes open (#8–9). These postural manipulations were designed to approximate stereotypical postures adopted during toe off and heel strike, key phases of gait.

All nine conditions were tested with a total of six C-FINE contacts (three per participant). Suprathreshold electrical stimulation paradigms were chosen after finding sensory detection thresholds via a forced-choice, two-alternative tracking paradigm^53^. Pulse width varied by contact from 80–200 μs, pulse amplitude varied between 0.8–1.2 mA, and pulse frequency was set at 20 Hz. The responses evoked by each C-FINE contact were evaluated 15 times per condition, except for one contact with participant LL02 that stopped responding to stimulation due to electrical connection issues unrelated to the experiment. For that contact (R1), we collected at least ten trials per condition. Each experimental session lasted approximately three hours, including time for breaks. Trials were randomized between different electrode contacts in each session. Testing for each contact was typically completed within three sessions, which were 1–6 weeks apart.

**Figure 3:**
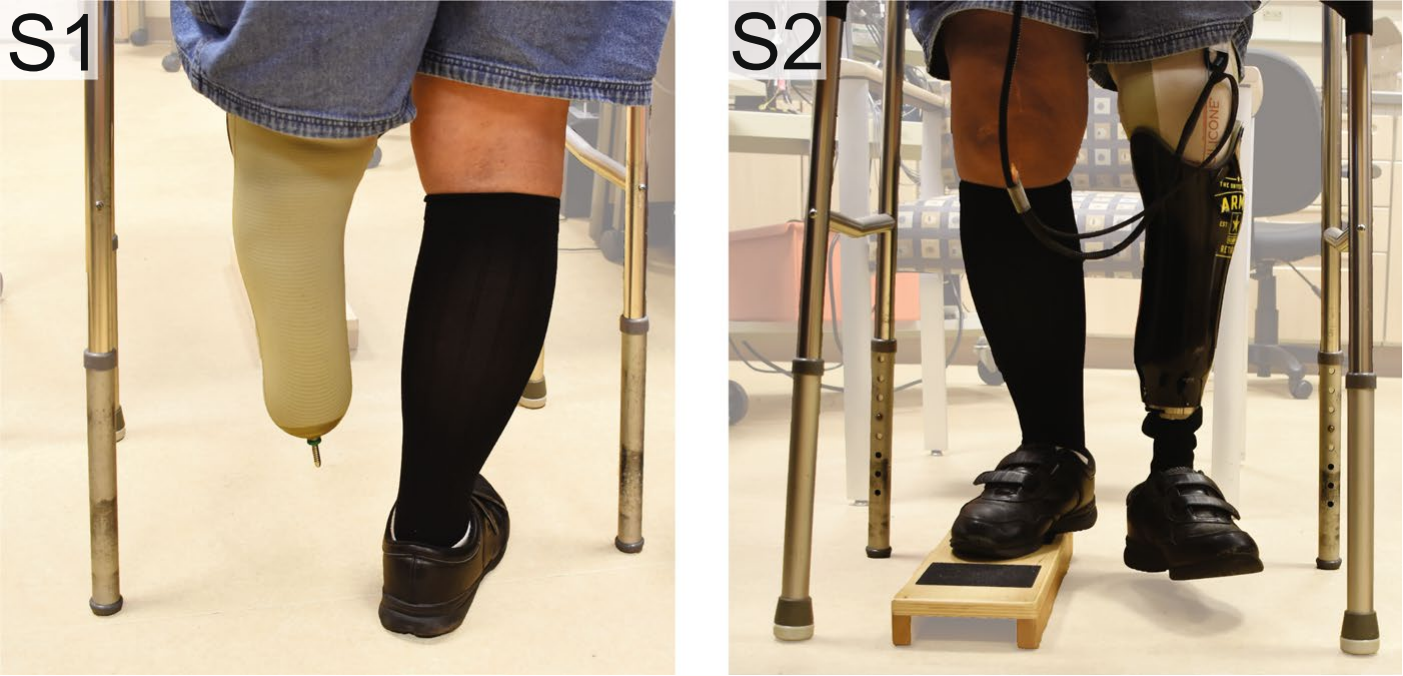
Participant LL01 demonstrates the two supplemental static standing conditions. The number in the top left corner of each photo represents the condition number described in Table 1. When testing contacts F1 and F2, the eyes were open. For contacts R2 and R3, the eyes were closed.

After observing differences in the reported percepts during the seated versus static standing conditions, potential causes for these differences were evaluated with additional testing. To evaluate if interactions between electrode contacts and the primary neural fibers activated by electrical stimulation were affected by posture, detection thresholds were collected four times per contact while participants were sitting, and again while they were standing. To determine if changes in percept size were due to the recruitment of additional neural fibers, two contacts were re-tested with a larger electric field induced by delivery of a higher stimulation level while participants were seated. We increased the charge until participants verbally reported the intensity to be double the initially reported level. We also hypothesized that neural fibers do not change their orientations with respect to C-FINE contacts due to changes in body position alone. To evaluate this, we re-tested four contacts while participants stood upright without their prostheses on, while holding onto a walker to maintain stability (condition #S1, Table 1 and Fig. 3). Finally, we hypothesized that cognitive expectations of foot-floor contact associated with donning the prosthesis affected reported percept size. To test this, each participant reported percept locations while standing on a wooden box with the intact leg and letting the prosthetic leg dangle in the air without contacting the ground (condition #S2, Table 1 and Fig. 3).

### Data analysis

All the collected electronic drawings were processed and analyzed using the Image Processing Toolbox in MATLAB. The toolbox helped to convert drawings into binary mask images, in which pixels for the reported percept areas were set to 1, and all pixels outside the area were set to 0. The foot diagram was divided into three regions of interest (ROIs) that represented the areas most frequently involved in gait and balance: the forefoot, midfoot, and rearfoot. A primary ROI was assigned to each contact. The ROI in which sensations were reported in the greatest number of the baseline trials was identified as the primary ROI.

**Figure 4:**
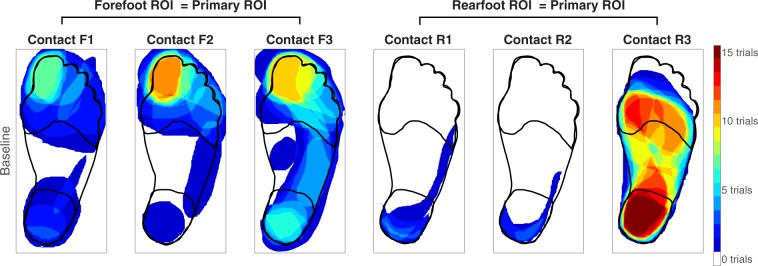
During the baseline condition, each participant sat with the prosthesis elevated while an electrode contact delivered stimulation to the nerve. Participants then drew the perceived location of the evoked percept on a blank diagram of the intact foot and leg, represented here as one heat map per contact. Red areas indicate regions that were drawn in all fifteen trials. The forefoot region of interest (ROI) was classified as the primary ROI for contacts F1-F3, and the rearfoot ROI was classified as the primary ROI for contacts R1-R3.

Inputs collocated with the primary ROI were classified as “congruent.” Using the baseline data, the three contacts from participant LL01 were classified as congruent with inputs about the forefoot (referred to as contacts F1-F3) and three from participant LL02 were congruent with inputs about the rearfoot (referred to as contacts R1-R3) (Fig. 4). In order to test the greatest spatial mismatch, incongruent inputs were applied to the rearfoot when the primary ROI was the forefoot, and vice versa.

In every trial, an activation percentage was assigned to each region of the foot based on how much of the region was covered by the percept drawn by the participant (Supplementary Fig. S1). The equation for activation percentage was the following:

$$Activation\,percentage=100\times \frac{area\,of\,percept\,within\,ROI}{area\,of\,ROI}$$

Activation percentages were calculated for the full plantar surface of the foot and each ROI. The mean and standard errors of these activation percentages are given in Supplementary Table S1.

### Statistical analyses

Paired t-tests with significance levels of α = 0.05 determined if the activation percentages in one condition were significantly different than the baseline condition. We split the electrode contacts into two groups based on their primary ROIs, therefore we grouped the forefoot contacts (F1, F2, F3) and rearfoot contacts (R1, R2, R3) together during statistical analyses. We compared the primary ROI between conditions and a combination of the two remaining ROIs (referred to later as ‘regions outside of the primary ROI’) between conditions.

During the comparisons of sitting versus static standing (conditions #2, #3, #S1, #S2), we analyzed the activation of the whole plantar foot surface because we did not add inputs to specific ROIs. Moreover, two-tailed tests were performed because we hypothesized that there would be no significant changes in percept size. In all congruent and incongruent conditions (#4–9), one-tailed t-tests were used to reflect our hypotheses that congruent information localizes percepts, and incongruent information causes percepts to spread. A one-way repeated measures analysis of variance (ANOVA) was used to compare the sensory detection thresholds between sitting and standing for all electrode contacts.

## Results

### Perceptual differences between sitting and static standing

**Figure 5:**
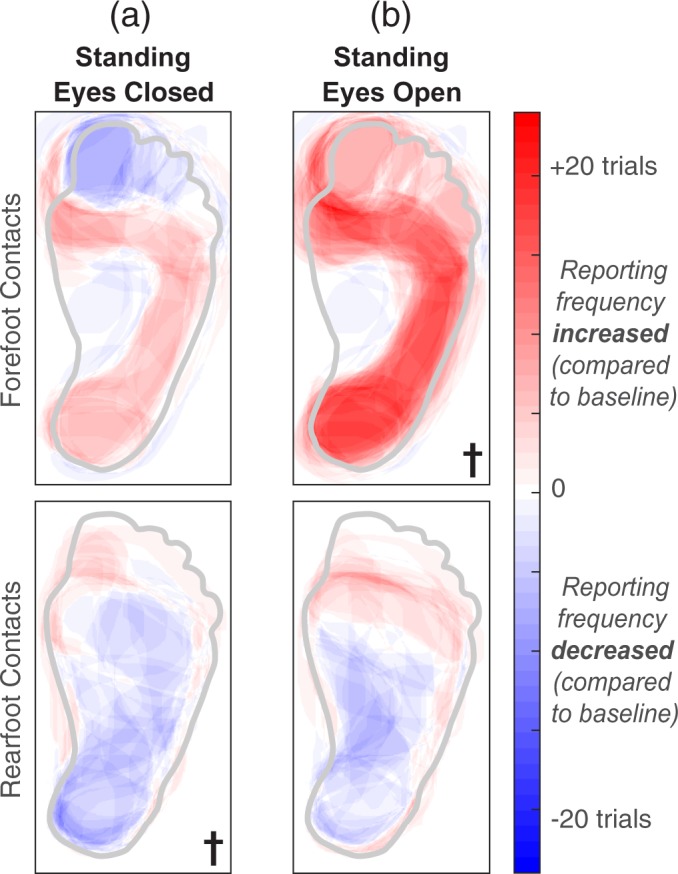
A generic healthy foot is outlined in grey. Shaded red areas indicate regions that were reported more than the baseline condition, and shaded blue regions represent a decrease in reporting compared to baseline. The † symbol indicates significant changes in percept reporting frequency and/or percept size over the entire plantar surface of the foot (two-tailed paired t-tests, p < 0.05). (**a**) Stimulation was delivered while participants stood upright with their eyes closed. (**b**) Stimulation was delivered while participants stood upright with their eyes open, looking down at their feet.

With respect to the baseline condition, activation in the entire plantar surface of the foot was significantly different during static standing (Fig. 5). Somatosensory percepts evoked by rearfoot contacts were different when the eyes were closed (condition #2, *p* = 0.031) and the percepts evoked by forefoot contacts were different when the eyes were open (condition #3, *p* < 0.001). For the rearfoot contacts, activation in the plantar surface of the foot decreased by 4 ± 2%. For the forefoot contacts, activation in the plantar foot surface increased by 20 ± 5%.

**Figure 6:**
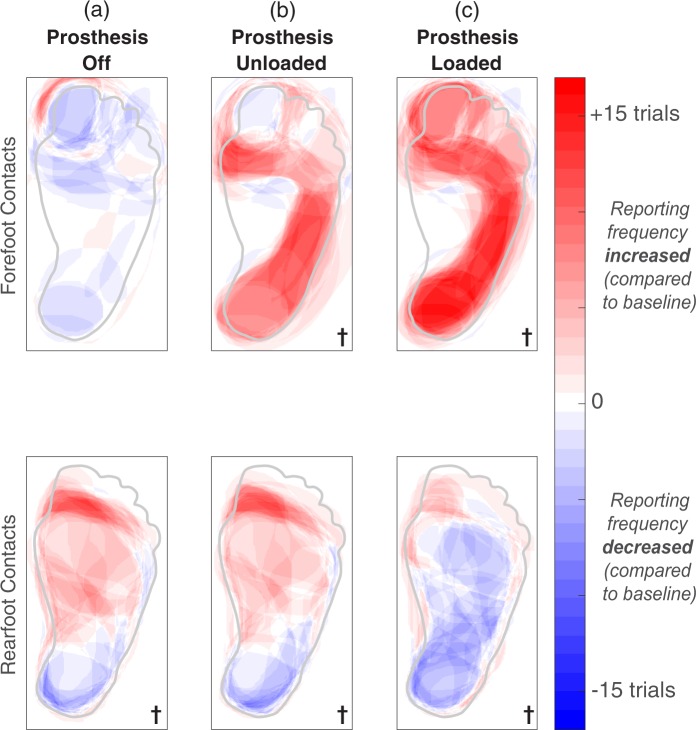
A generic healthy foot is outlined in grey. Shaded red areas indicate regions that were reported more than the baseline condition, and shaded blue regions represent a decrease in reporting compared to baseline. The † symbol indicates significant changes in percept reporting frequency and/or percept size over the entire plantar surface of the foot (two-tailed paired t-tests, p < 0.05). We re-tested contacts F1, F2, R2, and R3 in two supplemental conditions. When testing contacts F1 and F2, the eyes were open in all three conditions shown here. For contacts R2 and R3, the eyes were closed. (**a**) During the “prosthesis off” condition, electrical stimulation was delivered while a participant stood upright without wearing a prosthesis. (**b**) During the “prosthesis unloaded” condition, electrical stimulation was delivered while a participant stood on a wooden box with the intact leg and let the prosthetic leg dangle in the air. (**c**) Electrical stimulation was delivered while participants stood upright with their prostheses loaded. These results are also shown in Fig. 5, but repeated here to easily identify perceptual differences between standing conditions.

While participants stood upright without wearing their prostheses (condition #S1), only percepts evoked by rearfoot contacts were significantly different than the baseline condition (*p* = 0.013, Fig. 6a). In a post hoc two-tailed t-test that ungrouped the rearfoot contacts, there was no significant difference in activation percentage for contact R2 with respect to the baseline; the baseline percentage was 1 ± 1% and the condition #S1 percentage was 0 ± 0%. Therefore, while standing with the prosthesis off, the statistical effect for the rearfoot contacts was largely dominated by contact R3. While participants stood upright but did not load their prostheses on the ground (condition #S2), both groups of electrode contacts evoked significantly different percepts in the plantar surface of the foot (Fig. 6b) (*p* = 0.021 for the forefoot contacts, *p* = 0.004 for the rearfoot contacts). Activation percentages in the plantar surface of the foot increased for contacts F1, F2, and R3 and decreased for contact R2.

**Figure 7:**
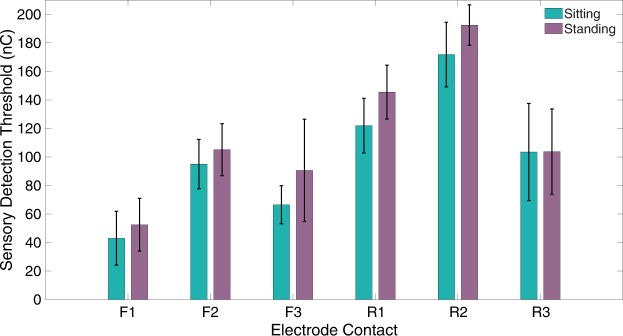
Sensory detection thresholds while participants sat down with their prostheses elevated (teal) or stood upright with their eyes open (purple). N = 4 trials per contact per posture. The black bars indicate standard deviation.

Sensory detection thresholds were not significantly different between sitting and standing. Average thresholds across all six contacts were 100 ± 45 nC while sitting and 115 ± 48 nC while standing (Fig. 7). We re-tested contacts F1 and F2 at a higher charge level and did not find a significant increase in percept area on the plantar foot surface (Supplementary Fig. S2).

### Perception during congruent inputs

**Figure 8:**
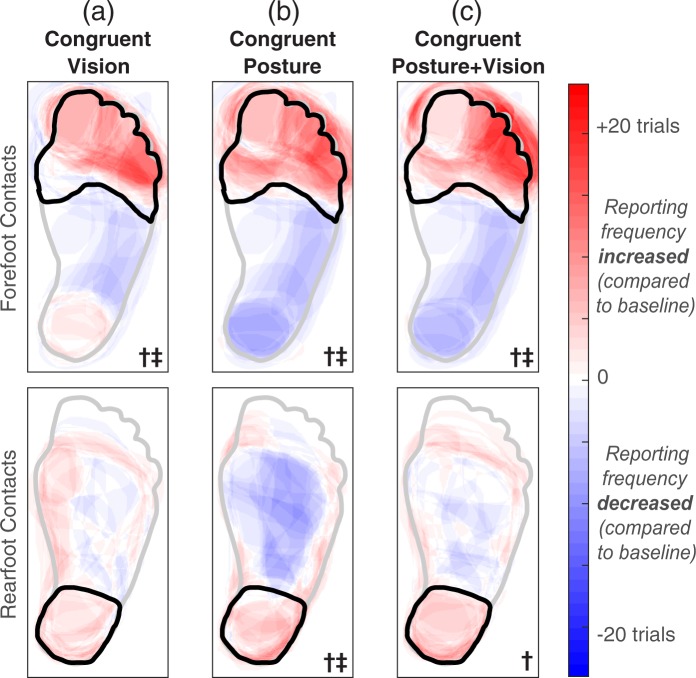
A generic healthy foot is outlined in grey, and the location of an added input is outlined in black. “Congruent” signifies that the experimenter touched the location of the primary ROI. The forefoot contacts (F1-F3) had a primary ROI in the forefoot, and rearfoot contacts (R1-R3) had a primary ROI in the rearfoot. Shaded red areas indicate regions that were reported more than the baseline condition, and shaded blue regions represent a decrease in reporting compared to baseline. The † symbol denotes significantly increased activation in the primary ROI, and the ‡ symbol indicates significantly decreased activation in regions outside of the primary ROI (one-tailed paired t-tests, p < 0.05). (**a**) During the conditions involving congruent visual inputs, electrical stimulation was delivered while participants sat and watched an experimenter apply a light touch to the primary ROI on the plantar surface of the prosthesis. (**b**) During conditions involving congruent postural manipulations with the eyes closed, electrical stimulation was delivered while participants stood upright and adopted a posture that applied a load to the location of the primary ROI. (**c**) Repeated condition *‘b’* with the eyes open.

The addition of congruent visual inputs (conditions #4 and #5) caused more localized percepts than the baseline condition for the group of forefoot contacts (Fig. 8a). Reports of percepts in the primary ROI increased in frequency and/or grew to cover more of the ROI (*p* = 0.003). Although electrical stimulation paired with congruent visual inputs led to increased activation of the primary ROI for the rearfoot contacts, the effect was not strong enough to have statistical significance (*p* = 0.066). For rearfoot contacts R1 and R2, the baseline percepts were primarily located on the side of the ankle with a few percepts reported on the heel. It is possible that the perception of percepts on the ankle overrode the percepts on the heel, interfering with the ability of visual inputs applied to the heel to act as “congruent” with electrical stimulation.

The addition of congruent postural manipulations (conditions #6 and #7) caused more localized percepts than the baseline condition for both groups of electrode contacts (Fig. 8b). Primary ROI activation increased (*p* = 0.004 for forefoot contacts and *p* = 0.046 for rearfoot contacts) and activation outside of the primary ROI decreased (*p* = 0.003 for forefoot contacts and *p* = 0.013 for rearfoot contacts).

During conditions with congruent visual inputs and postural manipulations (#8 and #9), percepts were more localized than the baseline condition for both groups of electrode contacts (Fig. 8c). Primary ROI activation increased for both groups of contacts (*p* = 0.006 for forefoot contacts and *p* = 0.035 for rearfoot contacts) and activation outside of the primary ROI decreased for the forefoot contacts (*p* = 0.005).

### Perception during incongruent inputs

**Figure 9:**
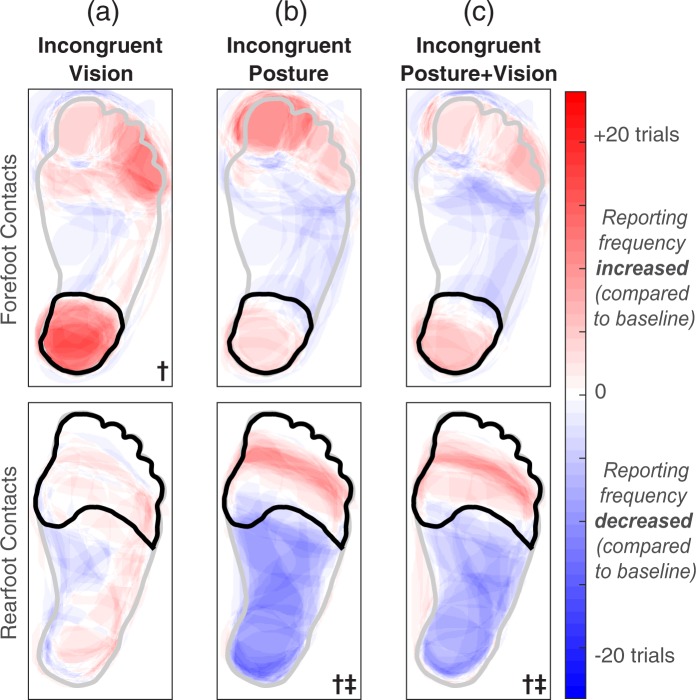
A generic healthy foot is outlined in grey, and the location of an added tactile input is outlined in black. “Incongruent” signifies that the experimenter touched a location outside of the primary ROI (forefoot contacts had a primary ROI in the forefoot, and rearfoot contacts had a primary ROI in the rearfoot). Shaded red areas indicate regions that were reported more than the baseline condition, and shaded blue regions represent a decrease in reporting compared to baseline. The † symbol denotes significantly increased activation in regions outside of the primary ROI, and the ‡ symbol indicates significantly decreased activation in the primary ROI (one-tailed paired t-tests, p < 0.05). (**a**) During the conditions involving incongruent visual inputs, electrical stimulation was delivered while participants sat and watched an experimenter apply a light touch to a region outside of the primary ROI. (**b**) During conditions involving incongruent postural manipulations with the eyes closed, electrical stimulation was delivered while participants stood upright and adopted a posture that applied a load away from the location of the primary ROI. (**c**) Repeated condition *‘b’* with the eyes open.

Incongruent visual inputs (conditions #4 and #5) led to an increase in activation in the ROI touched by the experimenter, which was outside of the primary ROI, for the forefoot electrode contacts only (*p* = 0.027, Fig. 9a). Conversely, incongruent postural manipulations produced a decrease in primary ROI activation in the rearfoot electrode contacts (Fig. 9b,c). This occurred in both the eyes closed (*p* = 0.001, conditions #6 and #7) and eyes open (*p* = 0.004, #8 and #9) conditions. Additional post hoc t-tests confirmed that this effect was again dominated by electrode contact R3. Contacts R1 and R2 did not evoke significantly different percepts between baseline and incongruent postural manipulation conditions, but contact R3 did (eyes closed *p* < 0.001, eyes open *p* = 0.003). The primary ROI for contact R3 was classified as the rearfoot from baseline data, but the entire plantar surface of the foot was frequently reported (parts of the forefoot ROI were reported in 41 ± 5% of trials, compared to 84 ± 2% for the rearfoot, which was the primary ROI). Though there were significant differences, they may not have been the result of truly “incongruent” inputs, but rather postural manipulations helping to focus attention on an alternate region that had fewer reported percepts than the primary ROI in the baseline condition.

## Discussion

Humans integrate multiple streams of information to develop an internal understanding of the external environment and their interactions with it^54–56^. Information from one sensory stream can affect perception of information from another, helping to reinforce or redefine ambiguous information^56,57^. In this study, we evaluated the perception of touch size and location by selectively manipulating the interplay between afferent somatosensory information, body posture, and vision.

### Expectations of foot-floor contact can impact the size of somatosensory percepts

Static standing affected percept location with respect to the baseline condition. While standing, percepts covered a smaller percentage of the foot surface for rearfoot contacts, and a larger percentage for forefoot contacts. These changes were most likely not due to any changes in nerve fiber recruitment. Sensory detection thresholds were not found to be significantly different between sitting and standing, which indicates recruited neural fibers did not move towards or away from the tested C-FINE contacts. However, it was still possible that the transition from sitting to standing would change the nerve cross-sectional geometry and consequently result in recruitment of additional fibers. To address this, while participants were seated, we increased the delivered charge density to broaden the electric field and recruit any smaller nearby fibers. However, we found that percept size on the plantar surface of the foot did not significantly increase in response to the elevated charge density, suggesting that increases in percept size while standing were not due to additional fiber recruitment. One limitation of this test is that we may not have increased charge enough to see an effect. However, increasing charge levels any further caused discomfort to the participant.

While standing without wearing the prosthesis, there were few differences in percept size compared to a seated position, indicating that simply changing body position did not affect fiber recruitment. While stimulation through one contact (R3) elicited a significant increase in percept area, percepts evoked by the same contact covered over half of the plantar surface of the foot during the baseline condition. Therefore, this contact may have been close to multiple fibers of a similar diameter, and even small changes in posture could have realigned nearby fibers and affected recruitment. In contrast, donning the prosthesis but keeping it unloaded while standing affected percepts evoked by both groups of electrode contacts. We suspect that this was a result of the individuals’ internal knowledge of limb length (length of the residual leg plus the prosthesis) and expectations of potential foot-floor contact while wearing the prosthesis.

### Percepts were focused by congruent information

Our results confirmed that the addition of at least one congruent source of information helped participants clarify the location of stimulation-induced somatosensory percepts. Though previous studies have shown that cognitive expectations influence tactile acuity^45^, the effects of postural manipulations were not well established. We found that postural manipulations, which are accompanied by an intrinsic understanding of the expected consequences of those manipulations, caused an increase in tactile sensitivity with respect to baseline. Though the addition of visual information localized stimulation-induced sensory percepts, just as natural somatosensation can be influenced by vision^28,29^, congruent postural manipulations had an even stronger effect.

Each form of congruent inputs was likely assisted by directing participants’ attention to the primary ROI. Previous studies on natural somatosensation have found that sustained spatial attention to one region of the body results in enhanced processing of tactile stimuli in that region over unattended regions^58^. For the case of tactile sensations elicited by electrical stimulation, attending to the primary ROI may have made it easier for participants to identify percepts in that location and to ignore percepts that occurred in unattended regions.

The results of the congruent scenarios tested with PNS could relax certain constraints in the implementation of somatosensory feedback in prostheses. Malleable percepts that migrate to functionally relevant locations can improve the fidelity and perhaps the ultimate utility of sensory neuroprostheses in locomotion. When developing sensory neuroprostheses, amputees who lost their limbs many years ago may have some initial trouble visualizing restored limb sensation and identifying the locations of evoked percepts. Although the brain representation of a missing limb is maintained over many years^59^, we have found that there appears to be an acclimation period between the first-ever percept elicited by PNS and the ability to express a clear and consistent percept referred to a missing body part^13^. We hypothesize that individuals receiving somatosensory neuroprostheses would likely benefit from a protocol applying congruent inputs to help localize percepts to functionally relevant locations and accelerate this acclimation process. This is analogous to how previous studies have exploited natural sensory illusions to achieve a desired perceptual effect by compensating for missing details^47^.

### Visual capture occurred for congruent and incongruent visual inputs

While congruent visual information localized the area of perceived touch, incongruent visual information broadened the location of the perceived touch for the forefoot contacts. Our findings corroborate prior reports on visual capture with natural somatosensation^38^: the location of stimulation-induced somatosensory percepts was affected by the location of visual inputs. Similar to past studies, for the rearfoot contact group, we found that if the mismatch between two inputs was too great, the illusion of congruency was not reached^60^ and perceived touch did not shift to include the location where touch was observed. Specifically, if plantar regions outside of the primary ROI were never reported in the baseline condition (i.e., the midfoot or forefoot for contacts R1 and R2), the perceived touch could not be shifted to these areas. It is also possible that visual capture could have occurred if the spatial mismatch was not as large between the visual input and stimulation-induced somatosensation^60^. An alternative explanation for our observations with incongruent visual inputs is that they were not completely incongruent. For example, for all three forefoot contacts, percepts were reported outside of the primary ROI in at least one of 15 baseline trials. Visual inputs likely directed the participants’ attention to percepts that were less perceptible during the baseline condition, enhancing them by attending to that location^58^. These results provide further evidence of a gaining system for establishing multisensory congruency^37^. In subsequent studies, our protocol for disassociating somatosensation from other stimuli could provide a unique framework to examine which regions of the brain are involved in establishing congruency between two inputs.

### When incongruent with somatosensation, postural manipulations prevented visual capture

Postural manipulations have a gating effect on the ability of visual information to influence the perceived location of stimulation-induced somatosensation. Although observed during conditions with incongruent visual inputs, visual capture did not occur for the majority of contacts when incongruent postural manipulations were also present. Though all experimental conditions involved static postures, a movement command had to be executed in order to adopt each posture. An internal copy of this motor command, called an efference copy, accompanies self-generated movement. An efference copy is then used to create an internal prediction of the movement’s sensory consequences^38,61^. When there is a discrepancy between the predicted and actual sensory information, an internal prediction model is updated^62^. Even when these sensory predictions were isolated from vision during conditions #6 and #7, incongruent postural manipulations still did not modulate stimulation-induced percepts for the majority of contacts. Combined with our observations on congruent postural manipulations, this suggests that expectations based on motor commands can reinforce the location of perceived sensations, but they do not alter percept location.

Previous work also hypothesizes that visual capture only occurs if a seen posture is proprioceptively feasible^38^. Moreover, visual capture of touch occurs for body image (how one’s own body is perceived), but not as strongly for body schema (which is involved in self-generated actions)^63,64^. It is possible that body schema is not as heavily influenced by visual capture due to the involvement of proprioceptive information^46,63^. The postural manipulations in this study incorporated proprioceptive information from the residual limb, which likely influenced visual capture. If the mismatch between postural manipulations and visual inputs had been less drastic, visual capture may have occurred. It would be interesting to investigate if these observations also occur during active movements combined with visual inputs and electrical stimulation.

In future studies, it would be informative to apply incongruent inputs at different locations around the leg to test the sensitivity of visual capture. The primary somatosensory cortex (S1) is somatotopically organized with a layout that broadly follows the layout of the body itself. The foot region of S1 neighbors the toe and leg regions of S1^65^. A previous study found that visual capture of touch occurs in accordance with the somatotopic organization of S1^34^. While participants viewed the hand, tactile discrimination thresholds improved on the hand and the face, but not the foot. The hand and face regions border each other in the somatosensory homunculus, but multiple regions separate the hand from the foot. It would be interesting to use peripheral nerve stimulation as a tool to identify exactly how far apart two stimuli can be before visual capture is disrupted.

In a survey on phantom limb pain, 80% of amputees reported that they had experienced phantom pain over a four-week period prior to the survey^66^. The exact cause of phantom limb pain is not yet well defined. One previous study suggests that phantom pain is the result of incongruence between an efference copy and afferent sensory information^67^, whereas other studies could not establish this link^68–70^. Incongruent conditions in this study did not induce any pain in our participants.

### Study design limitations

Although we used a unique experimental design to evaluate the effects of congruent and incongruent inputs on somatosensation, our study had certain limitations. Our findings could become more generalized if they are repeated in a larger group of amputees with more diverse demographics in age, sex, and amputation etiologies. At the time of this study, both participants received electrical stimulation-induced somatosensation in the laboratory for over a year and had a clear phantom perception of their missing limbs. They perceived electrical stimulation-elicited sensations as originating from their missing limbs, which was different from their general phantom perception. Future studies with a larger sample size could determine how stimulation-induced sensation is affected by the anomalies in phantom perception.

We did not expect significant variability in percept location between sessions because previous work has demonstrated that somatosensory percepts evoked by nerve cuff electrodes in amputees remain stable over the course of five months^13^. However, some trial-to-trial variability in reported percept location can be expected. For example, able-bodied individuals had an average localization error of 11.7 ± 2.3 mm when reporting the location of a physical tactile stimulus applied to the foot^71^. Tactile localization variability can be caused by a number of things, such as one’s attentional state^72^. Such variability would be random, however, rather than systematic like the changes in location we observed in the present study.

Additionally, the exact timing between stimulation-induced sensation and physically-applied touch likely had some variation due to the experimenter’s response time and movement planning. However, this delay was minimal compared to the length of stimulation-induced sensation. Human response time and movement planning is typically about 262 ms^73^, more than an order of magnitude smaller than the length of stimulation-induced sensation.

## Conclusion

Using peripheral nerve stimulation to evoke somatosensory percepts, we developed an experimental design that isolated afferent somatosensory information, postural manipulations, and vision. Using this disassociation method, visual inputs and postural manipulations were either congruent or incongruent with stimulation-elicited somatosensation. Compared to sitting, we found that standing upright may cause changes in percept area due to the cognitive expectations of weight bearing and foot-floor contact. Percepts could be focused by congruent visual inputs and/or congruent postural manipulations. We also demonstrated that visual capture occurred when visual information was incongruent with stimulation-induced sensation, which matches previous studies with natural somatosensation^38^. When incongruent with somatosensation, postural manipulations prevented visual capture. Furthermore, our results suggest that expectations based on motor commands can reinforce the location of perceived sensations, but they do not alter  percept location.

These characterizations of multisensory integration are important for somatosensory prosthesis development because current neural stimulation paradigms can only approximate the afferent signals from natural tactile stimuli. Our results suggest that the redundancy of multisensory inputs can improve perceptual precision and provide feedback in regions of the foot that are important for balance and locomotion.

## Supplementary information

Supplementary information

## Data Availability

The datasets generated during the current study are available from the corresponding author on reasonable request.
