# Supplementary material for: Visual inputs and postural manipulations affect the location of somatosensory percepts elicited by electrical stimulation
Source: Sci Rep. 2019 Aug 12;9:11699. doi: 10.1038/s41598-019-47867-1 (PMC6690924; doi:10.1038/s41598-019-47867-1)
Supplement: Supplementary file 1 — Supplementary information [file 41598_2019_47867_MOESM1_ESM.docx]

**Supplementary Figure S1:** **Activation percentage calculation.** An image of the plantar surface of the foot was divided into three regions of interest (ROIs): forefoot (outlined in teal), midfoot (outlined in orange), and rearfoot (outlined in purple). For each trial, an activation percentage was calculated for each ROI. In the example below, representative opaque blue percepts are drawn in the forefoot and rearfoot ROIs. Assuming this is contact #1, condition #1, and trial #1, the activation percentage of the forefoot ROI is 20%, midfoot ROI activation=0%, and rearfoot ROI activation=40%.

**Supplementary Figure S2:** **Increased charge while sitting.** A generic healthy foot and each region of interest are outlined in grey. Shaded red areas indicate regions that were reported more than the baseline seated condition, and shaded blue regions represent a decrease in reporting compared to baseline. **(a)** Contacts F1 and F2 were re-tested at increased charge levels while participants remained seated. There were no significant increases compared to the baseline seated condition. **(b)** Stimulation was delivered while participants stood upright with their eyes open, at the same charge levels as the baseline condition. These results are also shown in Figure 5b, but repeated here to easily identify perceptual differences between increasing the charge and standing upright.

**Supplementary Table S1: Activation percentages.** Experimental condition numbers from Table 1 are included in the top row. Activation percentages are listed for the full plantar surface of the foot (‘Full foot’) and the three regions of interest: forefoot (Fore’), midfoot (Mid’), and rearfoot (Rear’). The ‘forefoot contacts’ row lists the average of all forefoot contacts tested in each condition. The ‘rearfoot contacts’ row displays the average of all rearfoot contacts. For conditions #S1 and #S2, only contacts F1, F2, R2, and R3 were tested.

|  | **Baseline**  **(#1)** | **Standing,**  **eyes closed**  **(#2)** | **Standing,**  **eyes open**  **(#3)** | **Standing,**  **prosthesis off**  **(#S1)** | **Standing, prosthesis unloaded**  **(#S2)** | **Congruent visual inputs**  **(#4 forefoot contacts,**  **#5 rearfoot contacts)** | **Congruent postural manipulations, without vision**  **(#6 forefoot contacts,**  **#7 rearfoot contacts)** | **Congruent postural manipulations, with vision**  **(#8 forefoot contacts,**  **#9 rearfoot contacts)** | **Incongruent visual inputs**  **(#5 forefoot contacts,**  **#4 rearfoot contacts)** | **Incongruent postural manipulations, without vision**  **(#7 forefoot contacts,**  **#6 rearfoot contacts)** | **Incongruent postural manipulations, with vision**  **(#9 forefoot contacts,**  **#8 rearfoot contacts)** |
| --- | --- | --- | --- | --- | --- | --- | --- | --- | --- | --- | --- |
| **Contact F1** | Fore: 24+9%  Mid: 4+3%  Rear: 12+7%  Full foot: 15+6% | Fore: 36+10%  Mid: 7+4%  Rear: 16+9%  Full foot: 22+7% | Fore: 53+7%  Mid: 25+6%  Rear: 60+9%  Full foot: 44+6% | Fore: 24+7%  Mid: 4+4%  Rear: 6+6%  Full foot: 13+5% | Fore: 52+9%  Mid: 21+6%  Rear: 43+12%  Full foot: 39+8% | Fore: 47+7%  Mid: 1+1%  Rear: 5+5%  Full foot: 22+3% | Fore: 58+6%  Mid: 2+1%  Rear: 0+0%  Full foot: 26+3% | Fore: 45+7%  Mid: 0+0%  Rear: 0+0%  Full foot: 20+3% | Fore: 39+8%  Mid: 4+2%  Rear: 38+9%  Full foot: 26+5% | Fore: 48+6%  Mid: 4+3%  Rear: 24+10%  Full foot: 27+2% | Fore: 29+8%  Mid: 0+0%  Rear: 16+7%  Full foot: 16+3% |
| **Contact F2** | Fore: 36+6%  Mid: 3+2%  Rear: 5+4%  Full foot: 18+3% | Fore: 30+10%  Mid: 13+5%  Rear: 26+10%  Full foot: 23+7% | Fore: 58+8%  Mid: 20+6%  Rear: 39+11%  Full foot: 40+7% | Fore: 24+6%  Mid: 0+0%  Rear: 0+0%  Full foot: 11+3% | Fore: 33+8%  Mid: 16+5%  Rear: 26+11%  Full foot: 25+7% | Fore: 48+7%  Mid: 3+2%  Rear: 18+8%  Full foot: 25+4% | Fore: 49+7%  Mid: 0+0%  Rear: 0+0%  Full foot: 21+3% | Fore: 57+8%  Mid: 1+1%  Rear: 11+6%  Full foot: 28+4% | Fore: 55+8%  Mid: 10+4%  Rear: 42+10%  Full foot: 36+5% | Fore: 33+5%  Mid: 0+0%  Rear: 12+7%  Full foot: 17+2% | Fore: 31+6%  Mid: 0+0%  Rear: 14+8%  Full foot: 16+3% |
| **Contact F3** | Fore: 39+8%  Mid: 15+6%  Rear: 28+9%  Full foot: 28+6% | Fore: 34+9%  Mid: 12+5%  Rear: 34+10%  Full foot: 26+7% | Fore: 50+9%  Mid: 18+6%  Rear: 43+11%  Full foot: 37+7% |  | | Fore: 48+7%  Mid: 1+1%  Rear: 26+9%  Full foot: 26+3% | Fore: 43+7%  Mid: 2+1%  Rear: 6+5%  Full foot: 20+4% | Fore: 46+7%  Mid: 3+1%  Rear: 0+0%  Full foot: 21+3% | Fore: 36+6%  Mid: 6+4%  Rear: 38+10%  Full foot: 25+4% | Fore: 38+7%  Mid: 5+3%  Rear: 21+8%  Full foot: 23+4% | Fore: 41+7%  Mid: 7+5%  Rear: 35+9%  Full foot: 27+5% |
| **Forefoot contacts** | Fore: 33+4%  Mid: 7+2%  Rear: 15+4%  Full foot: 20+3% | Fore: 33+5%  Mid: 11+3%  Rear: 26+6%  Full foot: 23+4% | Fore: 54+5%  Mid: 21+3%  Rear: 47+6%  Full foot: 40+4% | Fore: 24+5%  Mid: 2+2%  Rear: 3+3%  Full foot: 12+3% | Fore: 42+6%  Mid: 19+4%  Rear: 35+8%  Full foot: 32+5% | Fore: 47+4%  Mid: 2+1%  Rear: 16+5%  Full foot: 25+2% | Fore: 50+4%  Mid: 1+0%  Rear: 2+2%  Full foot: 23+2% | Fore: 49+4%  Mid: 1+0%  Rear: 4+2%  Full foot: 23+2% | Fore: 43+4%  Mid: 6+2%  Rear: 39+6%  Full foot: 29+3% | Fore: 40+3%  Mid: 3+1%  Rear: 19+5%  Full foot: 22+2% | Fore: 34+4%  Mid: 2+2%  Rear: 21+5%  Full foot: 20+2% |
| **Contact R1** | Fore: 0+0%  Mid: 1+1%  Rear: 12+6%  Full foot: 3+2% | Fore: 0+0%  Mid: 1+1%  Rear: 5+5%  Full foot: 2+2% | Fore: 1+1%  Mid: 1+1%  Rear: 8+8%  Full foot: 3+3% |  | | Fore: 2+2%  Mid: 8+6%  Rear: 21+7%  Full foot: 8+4% | Fore: 1+1%  Mid: 3+2%  Rear: 11+5%  Full foot: 4+2% | Fore: 0+0%  Mid: 1+1%  Rear: 13+9%  Full foot: 3+2% | Fore: 3+2%  Mid: 5+4%  Rear: 16+6%  Full foot: 6+3% | Fore: 0+0%  Mid: 1+1%  Rear: 3+2%  Full foot: 1+1% | Fore: 2+2%  Mid: 1+1%  Rear: 8+6%  Full foot: 3+1% |
| **Contact R2** | Fore: 0+0%  Mid: 0+0%  Rear: 5+3%  Full foot: 1+1% | Fore: 0+0%  Mid: 2+1%  Rear: 18+8%  Full foot: 4+2% | Fore: 3+3%  Mid: 2+2%  Rear: 16+7%  Full foot: 5+3% | Fore: 0+0%  Mid: 0+0%  Rear: 0+0%  Full foot: 0+0% | Fore: 0+0%  Mid: 0+0%  Rear: 0+0%  Full foot: 0+0% | Fore: 4+4%  Mid: 8+6%  Rear: 22+8%  Full foot: 9+5% | Fore: 1+1%  Mid: 4+2%  Rear: 37+11%  Full foot: 9+3% | Fore: 2+2%  Mid: 8+5%  Rear: 39+10%  Full foot: 12+4% | Fore: 1+1%  Mid: 6+4%  Rear: 13+7%  Full foot: 5+3% | Fore: 2+2%  Mid: 1+1%  Rear: 10+7%  Full foot: 3+2% | Fore: 2+2%  Mid: 0+0%  Rear: 2+2%  Full foot: 1+1% |
| **Contact R3** | Fore: 41+5%  Mid: 61+6%  Rear: 84+2%  Full foot: 57+4% | Fore: 41+5%  Mid: 41+5%  Rear: 45+11%  Full foot: 42+4% | Fore: 50+4%  Mid: 42+6%  Rear: 62+9%  Full foot: 49+4% | Fore: 63+2%  Mid: 80+3%  Rear: 74+7%  Full foot: 71+2% | Fore: 64+2%  Mid: 75+5%  Rear: 71+7%  Full foot: 69+3% | Fore: 42+6%  Mid: 49+6%  Rear: 79+6%  Full foot: 52+5% | Fore: 28+5%  Mid: 35+7%  Rear: 79+4%  Full foot: 40+4% | Fore: 43+5%  Mid: 48+8%  Rear: 79+6%  Full foot: 52+5% | Fore: 42+5%  Mid: 51+7%  Rear: 78+3%  Full foot: 52+4% | Fore: 54+4%  Mid: 12+3%  Rear: 27+10%  Full foot: 33+3% | Fore: 55+4%  Mid: 26+5%  Rear: 47+11%  Full foot: 42+4% |
| **Rearfoot contacts** | Fore: 15+4%  Mid: 23+5%  Rear: 36+6%  Full foot: 22+4% | Fore: 15+4%  Mid: 16+3%  Rear: 24+6%  Full foot: 17+3% | Fore: 19+4%  Mid: 16+4%  Rear: 30+6%  Full foot: 20+4% | Fore: 32+6%  Mid: 40+8%  Rear: 37+8%  Full foot: 36+7% | Fore: 32+6%  Mid: 37+7%  Rear: 36+7%  Full foot: 34+7% | Fore: 17+4%  Mid: 23+5%  Rear: 42+6%  Full foot: 24+4% | Fore: 10+3%  Mid: 15+3%  Rear: 44+6%  Full foot: 19+3% | Fore: 17+4%  Mid: 21+5%  Rear: 48+6%  Full foot: 25+4% | Fore: 17+4%  Mid: 22+5%  Rear: 37+6%  Full foot: 23+4% | Fore: 21+4%  Mid: 5+1%  Rear: 14+5%  Full foot: 13+3% | Fore: 22+4%  Mid: 10+3%  Rear: 20+5%  Full foot: 17+3% |

**Supplementary Table S2: Statistical results for standing conditions.** The p-values and 95% confidence intervals of all t-tests performed on standing conditions are listed below. Blue text indicates significant perceptual changes (p<0.05). Two-tailed t-tests were performed on the full plantar surface of the foot only, not individual ROIs. The lower and upper boundaries of the confidence intervals are in parentheses.

|  | **Standing**  **Eyes closed (#2)** | **Standing**  **Eyes open (#3)** |
| --- | --- | --- |
| **Forefoot contacts**  **(F1, F2, F3)** | Full foot: 0.51  (-13.5%, 6.8%) | Full foot: <0.001  (-30.8%, -9.6%) |
| **Rearfoot contacts**  **(R1, R2, R3)** | Full foot: 0.031  (0.4%, 8.5%) | Full foot: 0.58  (-3.1%, 5.4%) |

**Supplementary Table S3: Statistical results for supplemental standing conditions.** The p-values and 95% confidence intervals of all t-tests performed on standing conditions are listed below. Blue text indicates significant perceptual changes (p<0.05). Two-tailed t-tests were performed on the full plantar surface of the foot only, not individual ROIs. The lower and upper boundaries of the confidence intervals are in parentheses.

|  | **Standing**  **Prosthesis off (#S1)** | **Standing**  **Prosthesis unloaded (#S2)** |
| --- | --- | --- |
| **Forefoot contacts**  **(F1, F2)** | Full foot: 0.23  (-3.0%, 11.8%) | Full foot: 0.021  (-29.2%, -2.5%) |
| **Rearfoot contacts**  **(R2, R3)** | Full foot: 0.013  (-11.9%, -1.5%) | Full foot: 0.004  (-9.3%, -1.9%) |

**Supplementary Table S4: Statistical results for congruent inputs.** The p-values and 95% confidence intervals of all planned comparisons performed using data from congruent input conditions. Blue text indicates significant perceptual changes (p<0.05). One-tailed t-tests were performed on the primary ROI to determine if there was a significant increase in percept size and/or reporting frequency. One-tailed t-tests were also performed on a combination of the two remaining ROIs (regions outside of the primary ROI) to determine if there was a significant decrease in percept size and/or reporting frequency. The confidence intervals are given in parentheses. Because all t-tests were one-tailed, each interval contains positive or negative infinity (abbreviated as ‘inf’).

|  | **Congruent visual inputs** | **Congruent postural manipulations (without vision)** | **Congruent postural manipulations (with vision)** |
| --- | --- | --- | --- |
| **Forefoot contacts**  **(F1, F2, F3)** | Increase in primary ROI: 0.003  (5.9%, Inf)  Decrease outside of primary ROI: 0.18  (-Inf, 2.4%) | Increase in primary ROI: 0.004  (6.5%, Inf)  Decrease outside of primary ROI: 0.003  (-Inf, -3.6%) | Increase in primary ROI: 0.006  (5.8%, Inf)  Decrease outside of primary ROI: 0.005  (-Inf, -2.9%) |
| **Rearfoot contacts**  **(R1, R2, R3)** | Increase in primary ROI: 0.066  (-0.6%, Inf)  Decrease outside of primary ROI: 0.73  (-Inf, 5.8%) | Increase in primary ROI: 0.046  (0.2%, Inf)  Decrease outside of primary ROI: 0.013  (-Inf, -1.5%) | Increase in primary ROI: 0.035  (1.1%, Inf)  Decrease outside of primary ROI: 0.49  (-Inf, 4.0%) |

**Supplementary Table S5: Statistical results for incongruent inputs.** The p-values and 95% confidence intervals of all planned comparisons performed using data from incongruent input conditions. Blue text indicates significant perceptual changes (p<0.05). One-tailed t-tests were performed on the primary ROI to determine if there was a significant decrease in percept size and/or reporting frequency. One-tailed t-tests were also performed on a combination of the two remaining ROIs (regions outside of the primary ROI) to determine if there was a significant increase in percept size and/or reporting frequency. The confidence intervals are given in parentheses. Because all t-tests were one-tailed, each interval contains positive or negative infinity (abbreviated as ‘inf’).

|  | **Incongruent visual inputs** | **Incongruent postural manipulations (without vision)** | **Incongruent postural manipulations (with vision)** |
| --- | --- | --- | --- |
| **Forefoot contacts**  **(F1, F2, F3)** | Decrease in primary ROI: 0.95  (-Inf, 20.2%)  Increase outside of primary ROI: 0.027  (1.2%, Inf) | Decrease in primary ROI: 0.87  (-Inf, 15.9%)  Increase outside of primary ROI: 0.66  (-6.1%, Inf) | Decrease in primary ROI: 0.53  (-Inf, 10.1%)  Increase outside of primary ROI: 0.63  (-6.1%, Inf) |
| **Rearfoot contacts**  **(R1, R2, R3)** | Decrease in primary ROI: 0.68  (-Inf, 6.9%)  Increase outside of primary ROI: 0.41  (-3.6%, Inf) | Decrease in primary ROI: 0.001  (-Inf, -10.7%)  Increase outside of primary ROI: 0.99  (-8.9%, Inf) | Decrease in primary ROI: 0.004  (-Inf, -6.2%)  Increase outside of primary ROI: 0.94  (-5.1%, Inf) |
